# The worsening transplant organ shortage in USA; desperate times demand innovative solutions

**DOI:** 10.12860/jnp.2015.20

**Published:** 2015-10-01

**Authors:** Bahar Bastani

**Affiliations:** ^1^Division of Nephrology, Saint Louis University School of Medicine. Medical director of kidney transplantation, Saint Louis University School of Medicine, Saint Louis, USA

**Keywords:** End-stage renal disease, Transplantation, Diabetes mellitus, Hypertension

**Implication for health policy/practice/research/medical education:**

The great success in the field of transplantation has made it possible to save many lives every year. Unfortunately, this success has been overshadowed by an ever-growing shortage of organs. The ever-widening gap between demand and supply has resulted in an illegal black market and unethical transplant tourism of global proportions. While there is much room to improve the Iranian model of regulated incentivized live kidney donation, the Iranian model could serve as an example for how other countries could make significant strides to lessening their own organ shortage crises.

End-stage renal disease (ESRD) is a growing health concern in the United States. In the past 3 decades there has been an exponential, 1000%, increase in the number of ESRD patients in the United States, from 60 000 patients in 1980 to 187 000 in 1990, to 393 000 in 2000, and 594 000 in 2010. The prevalence rate of ESRD has increased by greater than 600%, from 290 cases per million in 1980 to 1754 cases per million of population in 2010 (1).

The causes of ESRD in the United States are diabetes mellitus (38%), hypertension (25%), glomerulonephritis (14%), renal cystic disease (5%), and other causes (18%) (1). The cost of ESRD has increased by 850% over the past 2 decades, from 5 billion dollars per year in 1990 to 16.74 billion dollars in 1998, and 42.50 billion dollars in 2009 (of which 29.03 billion was paid by Medicare public funds, and 13.47 billion by private funds) (2). According to the United States Renal Data System (USRDS) report, in 2010 of the total Medicare spending of 522.8 billion dollars (3.95% of GDP), 32.9 billion dollars were allocated for ESRD patients (6.3% of the total Medicare budget), and thus, ESRD patients consumed 0.25% of the GDP of the United States (1).

Among the 3 modalities of renal replacement therapy, i.e., hemodialysis, peritoneal dialysis, and renal transplantation, the latter has proven to be the most lifesaving, with a significantly reduced morbidity and mortality and a much-improved quality of life. The 85.5% 5-year patient survival for renal transplant is more than twice the 35.8% 5-year patient survival rate for dialysis patients awaiting transplantation (2). According to the USRDS report in 2012, of the total ESRD patients in the United States, 65% were supported with hemodialysis, 5% with peritoneal dialysis, and 30% had a functioning renal transplant. The yearly Medicare spending for an ESRD patient per modality was $87 561 on hemodialysis, $66 751 on peritoneal dialysis, and $32 914 on transplantation, with a total average of around $70 000 per year for an ESRD patient (1).

Since the first renal transplant in 1954, there has been a steady growth in the number of renal transplants per year, up to the year 2006 that it peaked at 17095 cases, after which the number has slightly declined and plateau (Figure 1). The two sources of kidneys for transplantation are either a live donor or a deceased donor. In the year 2000 the number of live donors equaled and subsequently slightly exceeded the cadaver donors up until the year 2004, when the number of live donors had peaked at 6647 per year. From 2004 up to the present time the number of live donors has plateau and even declined while the number of deceased donors has exceeded live donors, so that in the year 2012 there were 5622 live donors and 7420 deceased donors providing a total of 16 487 kidneys (10 868 kidneys from deceased donors, and 5619 kidneys from live donors) (3). The best results of kidney transplant are from live donors with 5-year patient and graft survival rates of 90% and 80%, respectively. This is in contrast to deceased donor kidney 5-year patient and graft survival rates of 82% and 66.5%, respectively (1). However, since 2004, the number of live donors has plateau and even declined (Figure 1).

**Figure 1:**
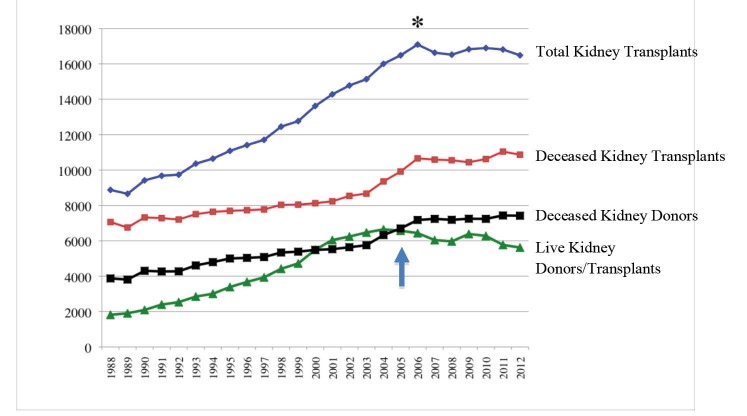
The prevalence of total kidney transplants (blue line), deceased kidney transplants (red line), deceased kidney donors (black line), live kidney donors and live kidney transplants (green line) in USA, from 1988 to 2012. * In the year 2006 the total number of kidney transplants peaked and after that it has plateau and even declined. In the year 2004 the number of live kidney donors/transplants peaked and after that it has plateau and even declined (Arrow).

Several possible explanations for this decline are as follows; (*a*) The United Network of Organ Sharing (UNOS) 2005 regulation prioritizing kidneys from deceased donors younger than 35 years to pediatric recipients, (*b*) The economic crisis since 2007 that forces people not to take any health risks in their lives, (*c*) The gradual decline in the health of general population in the United States (increasing percentage of population with diabetes, obesity, hypertension), (*d*) Increasing age of transplant candidates decreasing their chance of finding healthy older donors, (*e*) The new changes in donor selection criteria, and finally, (*f*) The more stringent regulatory oversight forcing transplant centers to adopt more conservative patient selection policies to improve their performance report card.

Whatever the causes may be, while the number of ESRD patients and those on the waiting list for transplantation has increased exponentially in the last decades, the number of available kidneys for transplantation has not increased in the past 7 years, and the number of live kidney donations has actually declined (Figure 2). In such environment, only 16% (16 487 patients) of those who needed a kidney (total of 101630 ESRD patients on the waiting list) received a transplant, another 7% (7363 patients) dropped off the waiting list because they either died or were too sick to be transplanted and over 70 000 Americans were left to suffer on dialysis at the end of 2012. The prospects are much worse for the older transplant candidates. Currently 63% of patients on the waiting list are older than 50 years and 20% are older than 65 years (3). While survival advantage has been shown across all age groups, including a near doubling of life expectancy among patients older than 60 years (4,5). Around half of kidney-transplant candidates on the waiting list who are older than 60 years die before receiving a deceased donor kidney (6).

**Figure 2:**
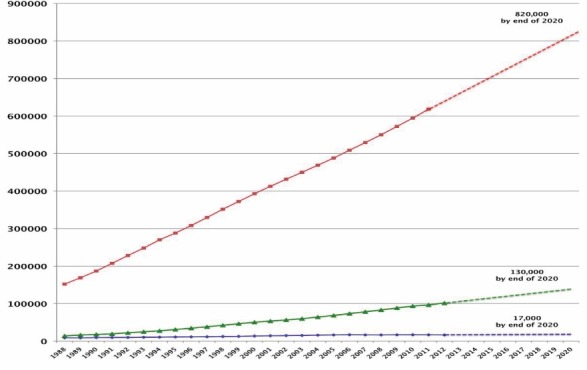
The prevalence of ESRD patients (red line), ESRD patients on kidney waiting list (green line), and renal transplants (blue line) in USA, from 1988 to 2012. The dotted lines extrapolate the prevalence by the year 2000.

In 1984, when the US Congress passed the law banning payment for organs, all bets were placed on a system of donated cadaver organs supplemented by altruistic living donations, however despite a very elaborate and advanced system of organ procurement. Today we are farther removed from the original premise. It is becoming more clear that current policies are far from sufficient to satisfy current and future needs and that this desperate time demands some drastic and innovative solutions.

One such solution would be an incentivized government or non-governmental organization (NGO) controlled model where donors would receive a fixed dollar amount as a gift of appreciation reciprocating their gift of a kidney to the community at large. This non-directed incentivized live donor program can supplement the existing altruistic directed live donation and the deceased donor programs. Such an incentive oriented NGO-controlled model of live kidney donation has been implemented in Iran and has been improved by the Iranian medical community over the past 25 years (7). The “Iranian model” has efficiently eliminated the black market for kidneys that exist in some other countries, and has eliminated the middleman who makes tens of thousands of dollars by arranging medically and ethically questionable kidney donations. Moreover, the transparency of the model has eliminated illegal, back-alley transplantations in shady hospitals by unqualified surgical and medical teams. It has also eliminated the poorly evaluated kidney sellers who could suffer or die for lack of medical care because of the fear of arrest and legal punishment, or sellers who would be cheated of the promised compensation without any legal resource.

In the Iranian model, the NGOs that supervise kidney donation and the transfer of the financial gift to the donors are primarily volunteers who themselves are kidney recipients or dialysis patients. These NGOs have standardized donor-recipient contracts, arrange for medical and psychological testing, and function as a gateway to a whole arsenal of social services available to both recipients and donors – everything from dental care to housing and small business loans – the donor’s fee is put in escrow by the NGO. Thus, kidney sellers are not cheated out of the money that they are promised, as commonly happens in countries where the black market is the only alternative to altruistic donation. The donors also receive from one to five years of free medical care that in some jurisdictions extends to the donors’ families, too. The “Iranian model” has particularly eliminated a transplant waiting list, and everyone who qualifies for a transplant can begin the process of arranging for a transplant by registering in the local NGO and waiting for a full health screen and matching of a potential donor. Meanwhile, the recipient should scrape together the funds to reciprocate the kidney gift with a nominal fee determined by the central NGO that is adjusted yearly based on the inflation rate. This nominal fee is kept in escrow by the NGO to be fully transferred to the donor at the time of discharge from hospital as a gift from the recipient. The donor also receives a gift of ten million Iranian Rials (equivalent of US $3000-$6000 buying power in 2008) from the government, as well as free health services, as mentioned above. Those who cannot afford the nominal “gift fee,” will remain on dialysis, which is provided freely to all ESRD patients while waiting for a kidney, through an ever-growing number of available cadaver kidneys. While the majority of people who sold their kidneys did so because they were in a financial predicament, but many also sold their kidneys simply to improve their standard of living, i.e., start a new business, purchase a long-term rental agreement, pay off a loan or house, or build an addition. Finally, Iran has not only solved the supply side of its kidney shortage, it is also the only country in the world with a waiting list of people who want to be live donors. Such a model implemented in the United States could reduce the current average 5-year wait time for a kidney, for those who are lucky to receive one, to just a few weeks or months that it takes to complete donor screening and donor/recipient matching.

I would like to make it clear that I am not suggesting that the West and the United States copy the Iranian model. We are clearly different cultures and our organ procurement system has evolved differently. Nonetheless, there are things we can learn from Iran’s 30 years of experience with compensated kidney donation. The most important lesson we can learn is that it is possible to make an incentive based approach to kidney procurement work. But, for such a system to be successful, it must provide donors with more than compensation for donation-related expenses. To overcome the organ shortage in the United States, we need to create a scheme that benefits both recipients and donors. We need an approach that reciprocates the gift of a kidney with a nominal gift that pays well beyond the expenses and the lost wages incurred in the process of donation. Moreover, I suggest that donors be provided life-long health insurance for the risks they have undergone to improve the livelihood of their fellow countrymen, in the same manner that the veteran receives. Compensating heroes is not a new idea – think of firefighters, the military and even women who donate eggs to help infertile couples have children. Since it is impossible to put a price on saving someone’s life, the nominal fee and lifelong health insurance would be reciprocating the heroic good act with a gift from society. It will serve merely as a bonus that would make it easier for a potential donor to commit to this heroic good deed. There should be no shame in one helping himself/herself while helping others. It is condescending to assume that financial instability causes people to lose their ability to make rationale choices, and to justify a paternalistic policy approach that does not protect the poor but denies them the opportunity to help themselves and others. Imagine what it would be like if donors receive not only enough compensation to cover donation related expenses, but to prevent foreclosure on the family home, to go to college, to start or expand a business, or climb out of debt – all the while, at the same time, saving someone in their community from suffering and dying on dialysis. Moreover, a system of compensated kidney donation can help contain medical costs, since it is much more expensive to keep a patient on dialysis than to do and maintain a transplant. The government could pay donors $50 000 and still save money in the long term. Thus, I propose three sources of kidneys for transplantation, (*a*) on going deceased donor kidneys providing to the best matches in the waiting list, (*b*) on going directed altruistic live kidney donors, who are fully compensated for their transplant-related expenses, and (*c*) a new program of government incentivized non-directed live kidney donation to a central NGO that directs the kidneys to the best deserving match in the waiting list. While the motivation of kidney donation in the latter group would be predominantly financial, and to a lesser degree altruistic, the recipients of those kidneys will be from all socioeconomic classes, and the expansion of the available kidney pool for transplantation would allow avoiding poor quality marginal organs, and would permit better selection and matching of the donors and recipients.

Considering that kidney donation has proven safe in long term donor follow up studies (8) and that the transplant community highly recommends altruistic live donations to even strangers, this proposal will be a win-win solution for all parties involved, i.e., the recipient who would receive a better quality kidney at a much shorter wait interval, the donor who would improve his/her financial situation, and the government that has already committed to the cost of caring all ESRD patients. Each patient who would receive a kidney transplant and be off hemodialysis would save the US government at least $50 000 per year.

We do not have to do anything as drastic as repealing the ban on organ sales. We can start by implementing a pilot project to test incentivized kidney donation on a regional basis and move on from there, depending on the results of those studies. At present, too many people, both potential kidney recipients and donors are suffering needlessly. It is time to open the door, even if just a crack and try to let people support themselves by help to others through compensated kidney donation.

## Conclusion

The ever-widening gap between demand and supply has resulted in an illegal black market and unethical transplant tourism of global proportions. While there is much room to improve the Iranian model of regulated incentivized live kidney donation, the Iranian model could serve as an example for how other countries could make significant strides to lessening their own organ shortage crises.

## Author’s contribution

BB is the sole author of the paper.

## Conflicts of interest

None.

## Funding/Support

None.
